# Extensive haemorrhagic necrosis of liver is an unpredictable fatal complication in dengue infection: a postmortem study

**DOI:** 10.1186/1471-2334-14-141

**Published:** 2014-03-14

**Authors:** SAM Kularatne, IVB Imbulpitiya, RA Abeysekera, RN Waduge, RPVJ Rajapakse, KGAD Weerakoon

**Affiliations:** 1 Department of Medicine, Faculty of Medicine, University of Peradeniya, Peradeniya, Sri Lanka; 2 Medical Unit, Teaching hospital, Peradeniya, Sri Lanka; 3 Department of Pathology, Faculty of Medicine, University of Peradeniya, Peradeniya, Sri Lanka; 4 Department of Veterinary Pathobiology, Faculty of Veternary Medicine and Animal Science, University of Peradeniya, Peradeniya, Sri Lanka; 5 Department of Parasitology, Faculty of Medicine and Allied Sciences, Rajarata University of Sri Lanka, Saliyapura, Sri Lanka

**Keywords:** Dengue fever, Liver cell necrosis, Acute liver failure, Autopsy study

## Abstract

**Background:**

Dengue infection carries a potential risk of death despite stringent management of plasma leak and haemorrhage. It appears that the extent of liver dysfunction determines the outcome.

**Methods:**

We present a postmortem study of five patients, died of dengue shock syndrome who had markedly elevated liver enzymes and irreparable circulatory failure.

**Results:**

All were females with a median age of 46 years (range 20–50 years). All had positive NS1 and IgM. Clinically, one patient developed severe degree of hepatic encephalopathy whilst three patients developed uncontrollable bleeding manifestations. Dengue virus was detected in three liver specimens by reverse transcription PCR. Histology of the liver revealed massive necrosis with haemorrhages in these patients with evidence of micro and macrovesicular steatosis with significant periportal inflammatory infiltrate. No significant ischaemic changes or necrosis was observed in the other organs.

**Conclusions:**

Severe haemorrhagic necrosis of the liver was the cause of death in these patients probably due to direct viral infection. Predilection for severe liver disease remains unknown. Therefore, it is prudent to think beyond plasma leak as the main pathology of dengue infection and attempts should be made to develop other treatment modalities to prevent and manage unforeseen fatal complications of dengue infection.

## Background

Dengue fever is an arboviral infection transmitted by mosquitoes of the genus Aedes, which is widely distributed in tropical and subtropical regions of the globe affecting up to 100 million people per year with 2.5 billion people at risk [1]. In Sri Lanka, dengue fever epidemics have been occurring with increased magnitudes but the worst epidemic was reported in 2009 with 35008 cases and 346 deaths of which 6638 cases and 51 deaths reported in the Central Province of Sri Lanka [2].

Dengue infection is caused by a single stranded RNA virus in the family Flaviviridae, which consists of 4 serotypes (DEN 1–4). Infection with any of the dengue virus serotypes may be asymptomatic in the majority of cases, but in symptomatic cases the severity could vary from dengue fever (DF) to dengue haemorrhagic fever (DHF) including dengue shock syndrome (DSS) [1]. The virus can infect many organs including liver, described from 1950s [3,4]. Over the years the pathophysiology of dengue virus infection had been extensively studied [5]. Studies suggest that three main systems play an important role in the pathogenesis of DHF/DSS: the immune system, the liver and endothelial cell linings of blood vessels [6]. Increased permeability of microvasculature and plasma leak is supposed to be the main dysfunction that leads to DHF and DSS [6]. With this understanding many management guidelines have been developed totally based on fluid resuscitation, resulting in reduced mortality [7]. Despite these efforts and stringent management, there is still a small proportion of patients die due to severe form of dengue infection all over the world.

Severe liver involvement is one of the risk factors identified in patients who die of dengue infection. In general, mild to moderate liver involvement with elevated liver enzymes is common in dengue infection [8] but, acute liver failure and hepatic encephalopathy are rare [9]. Many pathogenic mechanisms have been put forth to explain the liver involvement, but none has been fully conclusive. Therefore, further studies are needed to understand the exact mechanisms of liver damage.

The Teaching Hospital Peradeniya (THP) in the hilly Central Province of Sri Lanka maintains a prospective registry of all adult dengue admissions to the hospital since year 2000. The patients are managed according to the guidelines of WHO and the National Guideline of Sri Lanka [7]. The attempts have been made to keep the deaths to the minimum by using stringent fluid management. Yet five patients succumbed to dengue haemorrhagic fever and shock in spite of fluid resuscitation, intensive care and adequate supportive therapy. They all had extensive liver involvement. This necropsy based study aims to describe the extent of liver damage in dengue infection with its impact on the outcome and to think beyond fluid management as the sole treatment.

## Methods

### Confirmation of the diagnosis

All patients with fever admitted to the Professorial Medical Unit of THP were clinically assessed to identify dengue cases. The confirmation of the diagnosis was made using NS1 antigen in the first few days of fever and using serology later (dengue specific IgM and IgG ELISA). The clinical and laboratory data were recorded in a formatted data sheet and the regular daily assessments were recorded during the hospital stay. Depending on the severity of the infection, frequent monitoring of vital parameters were done and stringent fluid management was carried out. Autopsies were done in all deceased patients. All five cases included in this study presented during the period of year 2011 – 2013 and they qualified for the diagnosis of dengue infection as they had either positive NS1 antigen or positive serology or they had positive Reverse Transcription Polymerase Chain Reaction (RT-PCR) for dengue in tissues obtained at autopsy. Informed written consent for the autopsy study was taken from the next of kin and complete pathological postmortem was carried out in four cases whereas in one case, only core biopsies of liver, heart and kidneys were taken. This autopsy study is a part of dengue fever studies we are conducting, for which the ethical clearance has been obtained from the Ethics Committee, Faculty of Medicine, University of Peradeniya, Sri Lanka. Written consent for postmortem was obtained from next of kin of all deceased patients.

### Histopathological study

The autopsies were done by a pathologist with advanced experience in autopsy studies with the participation of a member from the medical team. Sections from all organs were sent to Department of Pathology, Faculty of Medicine, University of Peradeniya for histopathological examination. Formalin fixed tissues were processed, embedded in paraffin, and were cut into sections. The sections were stained with hematoxylin and eosin for histological examination. Reticulin stain was done for liver.

### RNA extraction, reverse transcription and PCR amplification

Fresh tissue samples from heart, lung, brain, kidney, pancreas, liver and spleen, and blood were collected at autopsies for molecular genetic analysis done at the Department of Veterinary Pathobiology of the Faculty of Veterinary Medicine and Animal Science, University of Peradeniya. RNA extraction was performed using RNA Mini Kit (Pure Link™ – ambion). RT- PCR was performed according to a previously described protocol [10].

Finally informed written consent from the next of kin for publication of these individual clinical details and the accompanying images was obtained.

## Results

### Clinical presentation

All five patients were females with an age range of 20 – 50 years (median, 46 years). Two patients were transferred from a regional primary care hospital, where as the other three patients directly admitted to THP. The two patients who were transferred were send due to persisting shock with fluid leak despite fluid resuscitation. On general appearance three of them were overweight and three had other co-morbid illnesses consisting of diabetes mellitus, hypertension and dyslipidaemia which were well controlled prior to this admission. The mean duration of fever on admission was 4 days (range 3–5 days) and by the time of death the total mean duration of the illness was 5 days (range 4–6). Three patients had diarrhoea and vomiting associated with fever, where as two patients had bleeding manifestations.

All clinical features on admission are summarized in Table 1. Four patients had low volume pulse on admission and blood pressures were unrecordable in two of them. The other patient, despite having a slightly high blood pressure on admission (160/100 mmHg) developed a rapid reduction in blood pressure (110/60 mmHg) within one hour of admission. Four patients had evidence of plasma leakage with pleural effusions and ascites on admission.

**Table 1:** Clinical features of patients on admission

	**Patient 1**	**Patient 2**	**Patient 3**	**Patient 4**	**Patient 5**
Gender	Female	Female	Female	Female	Female
Age (years)	34	20	49	50	46
Appearance	Average built	Overweight	Overweight	Overweight	Average built
Co-morbidities			DM	DM, HT	DM, Dyslipidaemia
Duration of fever on admission (Days)	3	4	5	3	5
Presenting complaints	Fever	Fever	Fever	Fever	Fever
	- Headache	- Myalgia	- Myalgia -	- Myalgia	- Vomiting
	- Vomiting	-Headache	- Diarrhoea	- Headache	- PV bleeding
	- Diarrhoea	- Menses	- Vomiting		
Cardiovascular system	Pulse – weak	Pulse-100 bpm	Pulse-100 bpm, low volume	BP – 160/100 mmHg which dropped to 110/60 mmHg	Pulse - weak
	BP-unrecordable	BP – 80/60 mmHg	BP - unrecordable		BP – 80/50 mmHg
Evidence of plasma leak	Ascites B/L Pleural effusion	Right pleural effusion	Clear lungs No ascites	Right pleural effusion	Ascites B/L Pleural effusion
Progress	Recurrent shock	Recurrent shock	- Hepatic encephalopathy	Uncontrolled bleeding	Bleeding
			- Bleeding		
Duration of illness at death (Days)	4	5	6	5	6

DM-diabetes mellitus, HT-hypertension, BP-blood pressure in mmHg, bpm-beats per minute, B/L-bilateral.

All five patients deteriorated despite proper management according to local guidelines [7]. Three patients developed uncontrollable profound bleeding manifestations and one patient developed grade 4 hepatic encephalopathy prior to death.

### Laboratory investigations

The Table 2 depicts the laboratory investigations during the hospital stay. It was not possible to do antibody titres in one patient but her NS1 antigen was positive. All five patients on admission had a very low platelet count of less than 20 × 10^9^/L (range, 4 – 17 × 10^9^/L). Severe degree of liver involvement was evident by the high aspartate aminotransferase (AST), alanine aminotransferase (ALT), prothrombin time and international normalized ratio ( PT/INR) and Bilirubin values in these patients.

**Table 2:** Laboratory investigation findings

	**Patient 1**	**Patient 2**	**Patient 3**	**Patient 4**	**Patient 5**
Platelets (×10^9^/L)	12	4	16	17	11
PCV %	40%	42	44.9	37	47.2
ALT (IU/L)	1473.9	400	3340	1771	
AST (IU/L)			11040	715	1207
INR	2.77	2.51	1.96	1.94	2.50
Bilirubin (μmolL)	45.55	37.35	51.74		
NS1 antigen	Positive	Positive	Positive	Positive	Positive
Dengue IgM	Positive	Positive	Positive	Positive	Not done

PCV – packed cell volume, ALT- alanine aminotransferase, AST – aspartate aminotransferase, INR- international normalized ratio.

### PCR analysis

The PCR detected dengue infection in multiple organs (Table 3). Type specific PCR found them to be DEN 1 serotype in all positive cases. Three patients had positive dengue PCR in their liver specimens. Positive results were seen in lung, kidney, pancreas and spleen as well. Myocardium was affected in one patient and PCR was negative in all brain tissue.

**Table 3:** Results of PCR analysis in tissue samples from different organs

	**Heart**	**Lungs**	**Brain**	**Kidney**	**Pancreas**	**Spleen**	**Liver**
**Patient1**	(-) ve	(+ ) Ve	(-) ve	(+) Ve	(+) Ve	(+) Ve	(+) Ve
**Patient 2**	Not done	Not done	Not done	Not done	Not done	Not done	(-) ve
**Patient 3**	(-) ve	(-) ve	(-) ve	(-) ve	(-) ve	(-) ve	(-) ve
**Patient 4**	(-) ve	(+) Ve	(-) ve	(+) Ve	(+) Ve	(+ )Ve	(+) Ve
**Patient 5**	(+) Ve	(+) Ve	(-) ve	(+) Ve	(+) Ve	(+) Ve	(+) Ve

(-) ve – negative.

(+ )Ve - positive.

### Macroscopic findings at autopsy (Table 4)

**Table 4:** Summary of macroscopic findings of organs

	**Patient 1**	**Patient 3**	**Patient 4**	**Patient 5**
**Fluid accumulation**	Ascitic – 1200 ml	Blood stained	Ascites – 1500 ml	Blood stained
	PlE – 1000 ml	Ascites –1800 ml	PlE – 800 ml	Ascites – 1000 ml
		PlE – 300 ml	PeE – 100 ml	PlE – 1500 ml
**Liver**	Enlarged Congested	Congested	Enlarged Congested	Congested
		Subcapsular haemorrage	Subcapsular haemorrage	
		Petechial haemorrage		
**Gastrointestinal tract**	Eso. and stomach	Stomach and mesentery	Stomach and mesentery	Stomach and SI
	- Congested	- Petechiae	- Petechiae	- Haemorrhagic Area
	- Pin point haemorrage	- Haemorrhagic spots	- Haemorrhagic Spots	
**Heart**	Normal	Increased adiposity	LVH Congested	Epicardial haemorrhagic patches
**Brain**	Normal	Normal	Normal	Normal
**Lung**	No haemorrhage	Congested	Trachea- Petichiae	Interstital pneumonia
		Petichiae	No haemorrhage	
**Kidney & supra renals**	Congested	Haemorrhage in both renal pelvices	Petechial haemorrhage	Congested
		Supra renal glands – petechial haemorrhgaes		

PlE – pleural effusion, PeE-pericardial effusion, Eso- oesophagus, SI-small intestine, LVH-left ventricular hypertrophy.

Macroscopic examination of multiple organs was done in four patients. Patient number 2 had only histology of core biopsy of liver, kidney and heart. Macroscopically all other cases had enlarged and congested liver. Two specimens revealed subcapsular haemorrhages with multiple petechiae on the surface of the liver. All patients showed evidence of fluid leakage with pleural effusions and ascites with one patient additionally having a significant pericardial effusion. None of the organs showed macroscopic evidence of ischaemic changes or evidence of necrosis.

### Histopathological analysis (Figures 1, 2, 3, 4, 5, 6, 7 and 8)

**Figure 1:**
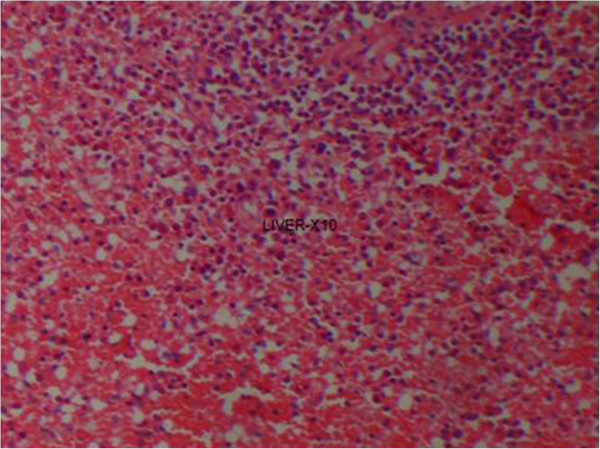
(Case 1) – H & E section of liver showing extensive haemorrhagic necrosis of liver parenchyma.

**Figure 2:**
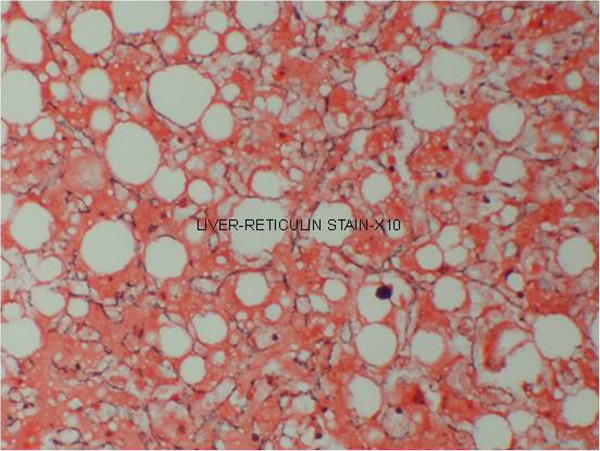
(Case 1) – Reticulin stain of liver with collapsed reticulin framework.

**Figure 3:**
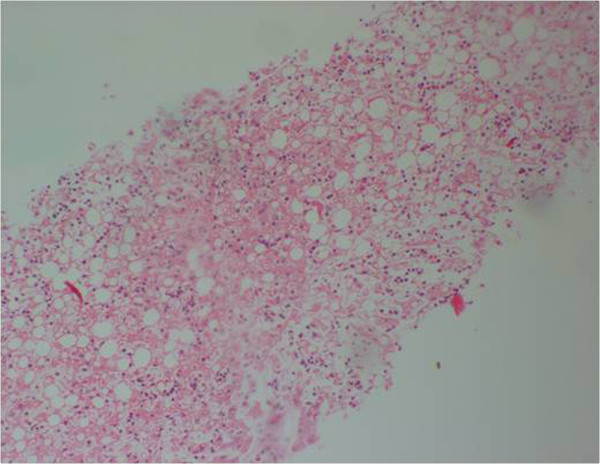
(Case 2) – H & E stain of Core biopsy of liver shows confluent necrosis.

**Figure 4:**
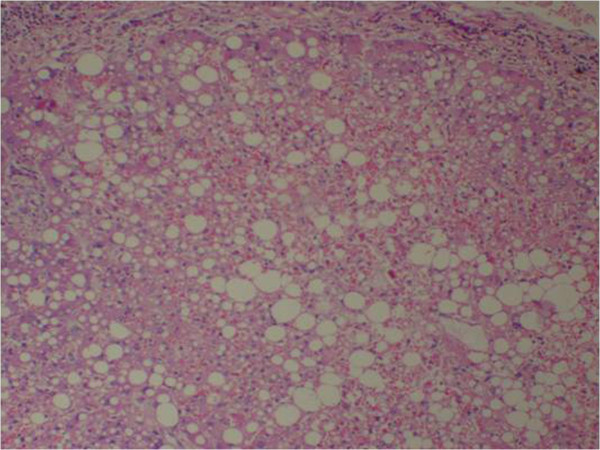
(Case 3) – H & E stain of liver showing extensive haemorrhagic necrosis with surviving hepatocytes reveal macro and micro vesicular fatty change.

**Figure 5:**
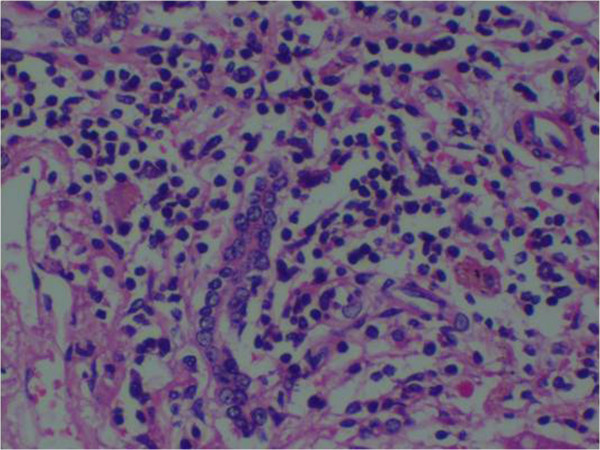
(Case 3) – H & E stain of liver showing lymphocytic infiltrate of the portal tract.

**Figure 6:**
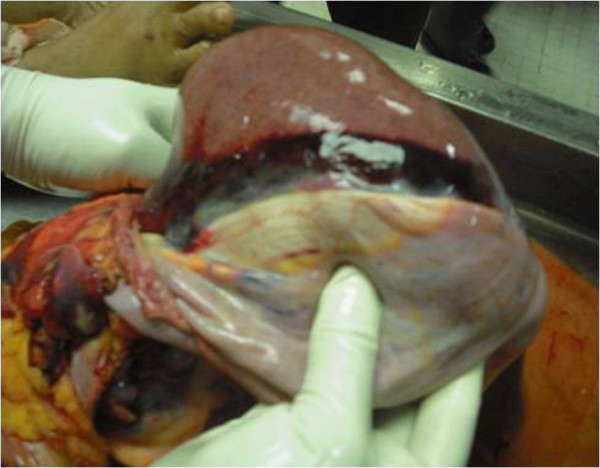
(Case 4) – Macroscopy of liver showing subcapsular haemorrhage.

**Figure 7:**
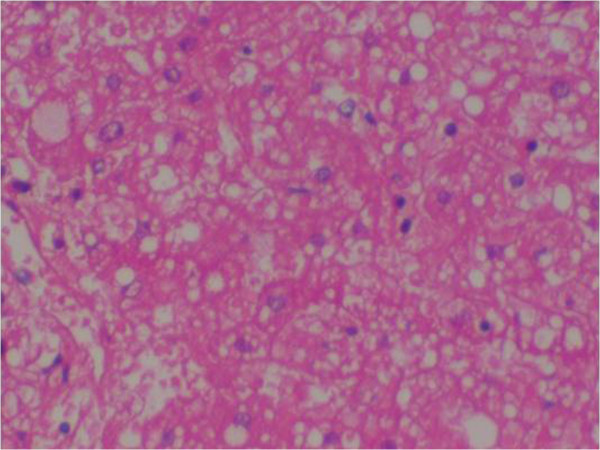
(Case 4) – Microscopy of liver H & E stain showing extensive necrosis.

**Figure 8:**
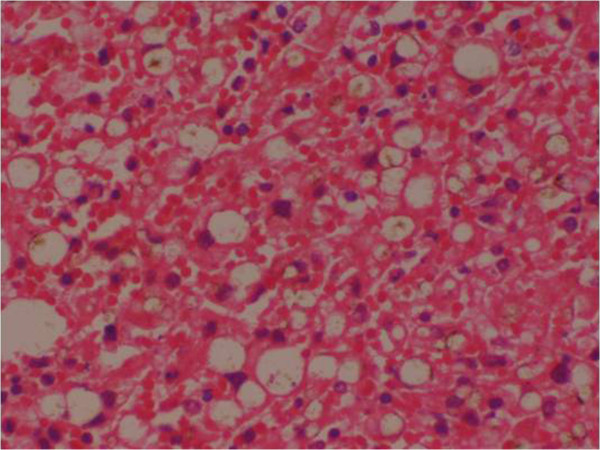
(Case 5) – Microscopy of liver H & E stain showing distortion of hepatic architecture by a confluent haemorrhagic necrosis.

Histology of the liver was almost same in all five specimens which showed distortion of liver architecture, red cell extravasation with collapse in the reticulin frame work and disintegration of hepatocyte nuclei suggestive of massive liver cell necrosis. These changes were predominantly seen in zone 1,2 and periportal regions. Micro and macrovesicular steatosis was also evident in these specimens. Acute inflammatory mononuclear cell infiltrates were predominantly seen in the periportal region with two specimens showing evidence of bridging necrosis with one specimen showing evidence of piecemeal necrosis.

Other organs such as the kidneys, suprarenal glands, pancreas, lung and heart predominantly showed evidence of congestion. None showed evidence of ischaemic changes or necrosis. Lungs showed evidence of interstitial pneumonitis suggestive of viral infection in patient 5. Brain specimens were all histologically normal.

## Discussion

Over a period of three years, a single tertiary care centre experienced five deaths due to dengue infection in Sri Lanka. Necropsies revealed extensive haemorrhagic liver necrosis as the main pathology behind these fatalities. Detection of viral genetic material by PCR indicated viral invasion of liver and causing direct liver damage. Simultaneously, infection was found in the other organs as well except brain. Clinically, these patients had unstable vital parameters and failed the resuscitation despite prompt fluid therapy and supportive care. Except for many fold of raised hepatic transaminases, severity of hepatic involvement was not apparent clinically. However, autopsy study unraveled the extent of hepatic necrosis in these patients. All these patients had erratic and unpredictable plasma leak, bleeding tendency and hypotension when compared to most of the cases of DHF which were amenable to management with meticulous fluid therapy. One limitation of the study was that we were unable to perform a complete autopsy study in one patient due to limitation of consent.

Severe to fulminant hepatitis tends to occur more often in DHF or DSS compared to classic dengue infections [11]. Unlike conventional viral hepatitis, in dengue infections the level of AST is higher than that of ALT [12]. It has been suggested that this may be due to excess release of AST from damaged myocytes during dengue infections [12]. Significantly higher elevations of AST and ALT were observed in patients with episodes of bleeding [12]. The five cases discussed here showed clinical, biochemical as well as pathological evidence of severe liver involvement. The elevation of transaminases is usually less than five-fold greater than upper limit of normal [13] in DF where as in these patients they were more than 10 times higher with an elevated INR.

The mechanism of hepatic involvement and hepatocyte damage in dengue fever is poorly understood. The histopathological findings of fulminant hepatitis associated with dengue haemorrhagic fever is often characterized by hepatocellular necrosis, typically localizing to zone 1 and 2 of the hepatic plate, a cellular inflammatory infiltrate and fatty changes. Councilman bodies are frequently present, and may represent necrosis around viral particles [14]. It is well established that most viral infections cause damage by an interplay of direct infection and concomitant host response, evidenced by up-regulation of cytokine production, notably TNFα, IL-2, IL-6, IL-8 and other chemicals [15,16].

Some literature suggest that liver damage in DHF is mainly due to viral replication in hepatocytes, and cytokine related damage due to adverse consequence of dysregulated host immune responses against the virus [17]. A dengue virus-specific CD4+ and CD8+ T cells may cause cytolysis. The dengue virus targets both the hepatocyte as well as kupffer cell and following internalization induces tumor necrosis factor-related apoptotic damage. The virus enters the hepatocytes and kupffer cells by phagocytosis and receptor-mediated endocytosis, respectively [18]. The main feature is the early generation of a unique cytokine, human cytotoxic factor (hCF) that initiates a series of events leading to a shift from Th1-type response in mild illness to a Th2-type response resulting in severe DHF. The shift from Th1 to Th2 is regulated by the relative levels of interferon-gamma and interleukin (IL)-10 and between IL-12 and transforming growth factor-beta, which showed an inverse relationship in patients with DF [19]. It is also noted that hepatosplanchnic venous pooling and/or dysfunction occur and correlate with the severity of circulatory derangement and shock in patients with DHF [20]. There was a report of a case of liver failure from dengue virus infection with reversal of portal venous blood flow which postulated that hepatic sinusoidal obstruction coupled with shock might be the underlying mechanism of liver failure in this disease [21].

The four DENV serotypes (1, 2, 3, and 4) have been co-circulating in Sri Lanka for more than 30 years with dengue epidemics occurring since 2009 due to DEN-1 [22]. All four serotypes have been associated with dengue-related fulminant hepatitis [23]. DEN-1 and DEN-3 seem to have more prominent liver affinity [24]. In addition, with another study, DEN-2 or DEN-3 viruses were recovered from the livers of 5 of 17 fatal cases [25]. We found DEN-1 as the serotype causing extensive hepatic necrosis in these patients.

We have considered many factors as contributory causes for the development of acute liver cell necrosis in these patients. The main factors being direct viral effect on liver cells, adverse consequence of dysregulated host immune responses against the virus, prolonged shock and haemorrhage, metabolic acidosis, intake of drugs during the early stages of the illness such as acetaminophen and pre-existing liver diseases such as fatty liver. Despite all these factors contributing, presence of the viral antigen in the liver (positive PCR), prominent inflammatory cells in liver, prominent damage seen in the periportal as well as zone 1 and 2 and the absence of ischaemic or necrotic changes in any of the other organs indicate that the liver damage was mainly due to the dengue virus infection rather than the other contributory causes. One would expect to have predominant changes in zone 3 as well as a less prominent inflammatory cell response if it were mainly due to ischaemic necrosis as a result of prolong shock. Studies indicate that other predisposing factors such as race, diabetes and sickle cell anaemia [26] can contribute to the liver damage. Three of above study patients were diabetics which may have contributed for fatty changes in liver. Therefore it is vital in the management to avoid or control the above mentioned factors occurring or take rapid steps to correct them as soon as they are identified. Another observatioin was the significant steatosis in all liver samples which could be due to a result of the infection itself or the other possibility of premorbid fatty liver disease in these patients putting them at higher risk of developing liver cell necrosis should be strongly suspected.

It was interesting to note that four out of the five patients presented with low blood pressure very early in the disease. It could be that this is the natural course of DSS with evidence of fluid leakage giving rise to hypovolaemic shock. At the same time with such early presentation (mean 4 days) with such severe disease, it is possible that the severe liver cell necrosis was the primary damage with subsequent low blood pressure as a consequence to that. The pathophysiology of hypotension in patients with fulminant hepatic failure was explained by Trewby et al. [27] where it was mentioned that the hypotension could be due to both a central as well as a peripheral effect. Peripheral vasodilatation may be a consequence of the appearance of vasoactive compounds in the circulation which may fail to be metabolised by the necrotic liver, or are released from it. Alternatively they postulated the presence of metabolic insult to the vasomotor center resulting in a low blood pressure.

In one study mortality and morbidity associated with fulminant hepatitis were higher in those with circulatory failure, severe thrombocytopenia and spontaneous bleeding, with deaths in these cases occurring in the first week of hospitalization which is also consistent with our study findings. In these instances, the fatality rate were as high as 12–44% [28,29]. There is no specific therapy for fulminant hepatitis in dengue except for supportive care. Better outcomes for DHF/DSS with fulminant hepatitis occurred in hospitals with more experience in managing such conditions, with case fatality rates as low as 0.2% [28]. In our cases, supportive care was instituted in the high dependency unit and intensive care given as early as possible. However, case studies of this nature intend to open up new research ideas to identifying the mechanisms of liver involvement in severe dengue infection and to discover therapeutic options. There is limited research that has been carried out to see whether treatment with drugs such as N-acetylcysteine (NAC) which is used in acute liver failure would be of benefit if given early in the course of the illness, to patients with liver involvement with dengue fever [30].

## Conclusion

Severe hepatic necrosis is an infrequent phenomenon seen with dengue haemorrhagic fever but when present carries a high mortality rate. It is therefore important to identify at risk individuals early and take steps to prevent them progressing into hepatic failure. It is also important to consider dengue haemorrhagic fever as one of the differential diagnosis for acute liver failure especially in endemic and epidemic areas of dengue. Even though, fluid management is the mainstay of treatment in dengue infection, attempts should be made to develop other treatment modalities to prevent and manage unforeseen fatal complications of dengue infection.

## Abbreviations

DEN: Dengue virus serotype; DF: Dengue fever; DHF: Dengue haemorrhagic fever; DSS: Dengue shock syndrome; THP: Teaching hospital peradeniya; RT-PCR: Reverse transcription polymerase chain reaction; AST: Aspartate aminotransferase; ALT: Alanine aminotransferase; PT/INR: Prothrombin time and international normalized ratio.

## Competing interests

The authors declare that they have no competing interests.

## Author contributions

SAMK conceived the idea. SAMK, IVB, RAA, KGADW and VJR recorded clinical data. RNW did autopsies and histopathological studies. SAMK, KGADW, IVB and RAA drafted the manuscript. VJR did PCR analysis. All the authors read and approved the final version of the script.

## Pre-publication history

The pre-publication history for this paper can be accessed here:

http://www.biomedcentral.com/1471-2334/14/141/prepub
